# Advancing Health Equity through ‘One World for Health’: Insights from the Seventy-Eighth World Health Assembly

**DOI:** 10.34172/hpp.45247

**Published:** 2026-06-06

**Authors:** Bawa Singh, Vijay Kumar Chattu

**Affiliations:** ^1^Department of South and Central Asian Studies, School of International Studies, Central University of Punjab, Bathinda, India; ^2^Department of Public Health, Health Administration & Information and Health Sciences, College of Health Sciences, Tennessee State University, Nashville, TN 37203, USA; ^3^ReSTORE lab, Department of OS & OT, Temerty Faculty of Medicine, University of Toronto, Toronto, ON M5G 1V7, Canada; ^4^United Nations University Institute on Comparative Regional Integration Studies (UNU-CRIS), Potterierei 72, 8000 Bruges, Belgium

**Keywords:** World health assembly, Health equity, Pandemic, Universal health coverage, Health technology, Health financing

## Abstract

This perspective aims to 1) examine the WHA78’s contributions to health promotion, equity, and analyze their implications for reducing disparities, and 2) propose actionable strategies to navigate implementation challenges and emphasize global solidarity in the achievement of ‘health for all’. The WHA78 has prioritized social determinants, community empowerment, equitable health access, and climate-resilient health systems. Strengthening health systems and addressing equity can be further complemented by the Pandemic Treaty’s enforceable compliance mechanisms. Further, the WHA allocated 20% of pandemic-related products to the WHO to address the needs of low-income countries. Strengthening regional and international health cooperation through health diplomacy, promoting sustainable health systems, sharing digital infrastructure, and fostering local/regional pharmaceutical manufacturing are vital to turning WHA78’s commitments into equitable outcomes.

## Introduction

 The 78th World Health Assembly (WHA78), convened under the theme “One World for Health,” marked a pivotal moment for advancing health promotion equity. It is a core principle of the 1986 Ottawa Charter for Health Promotion^1^ that emphasizes reducing disparities, empowering communities, and addressing social determinants of health. The WHA 78, held amidst the global challenges, including the United States’ planned withdrawal from the World Health Organization (WHO)^2^ and a $600 million budget shortfall.^3^ Notwithstanding these challenges, the key outcomes of WHA78 included the historic Pandemic Agreement, resolutions on health financing, controlling air pollution, emphasis on traditional medicine, and protections against exploitative marketing of breastmilk substitutes. These initiatives have taken on equity, social justice, and community-driven health solutions.

*WHO Financing:* The WHA78 was held on 19-27 May 2025 in Geneva (Switzerland), where representatives from all 194 WHO Member States participated to address ongoing critical health challenges and establish future health priorities.^4^ The adoption of the WHO Pandemic Agreement was one of the important agendas of the WHA, and the passing of the same after three years of negotiations. The agreement is intended to improve global pandemic prevention, preparedness, and response, based on the ‘One Health Approach’ and the principle of ensuring equitable access to health technologies. Another major agenda item covered was strengthening the WHO financing. The WHA member states were committed to increasing the assessed contributions. It also supported the newly introduced WHO Investment Round to ensure more flexible and predictable funding for the 2025–2028 strategic plan.

*Universal Health Coverage (UHC):* UHC was one of the important agendas of WHA78, with resolutions underscoring accelerated actions to improve equitable access to healthcare, especially for marginalized and vulnerable sections of the population.^5^ The Assembly advocated for increasing the health financing and investments in primary healthcare and health workforce development. They identified essential priorities for the Secretariat to support in the strategic plan leading up to the high-level meeting on universal health coverage in 2027. These priorities encompassed health system reforms via a primary healthcare framework, health financing and financial protection, addressing gaps in the health and care workforce, and utilizing data, digital health, and artificial intelligence, along with tailored strategies, especially in fragile, conflict-affected, and vulnerable environments.^6^

*Climate Change and Antimicrobial Resistance:* At the 28th Conference of the Parties (COP28) to the United Nations Framework Convention on Climate Change (UNFCCC), the 2023 event included the first-ever health-themed day. Following years of WHO leadership on health and climate change, addressing climate change was declared the top strategic priority in its Fourteenth General Programme of Work, adopted in May 2024.^7^ Concomitantly, climate change and its drastic impacts on health have also been recognized as persistent, critical issues. A resolution was passed advocating the development of climate-resilient health systems and supporting the most vulnerable countries in addressing the health effects of climate change. Another critical issue, antimicrobial resistance (AMR), was also on the list. The Assembly also reviewed advancements in assisting countries to prevent infections; guarantee universal access to high-quality, affordable diagnostics and appropriate treatment; bolster surveillance, research, and innovation; and improve awareness, governance, and financing related to antimicrobial resistance.^8^

*Maternal Health and Sexual & Reproductive Health:* The women, children, and adolescents’ health (WCAH) was another important dimension that has remained on the priority list. The Assembly reiterated its commitment to providing equitable access to maternal, neonatal, and child health services. It called for actions on the sexual and reproductive health rights of women, particularly in vulnerable and marginalized countries.^9^

*Strengthening Emergency, Critical, and Operative Care:* WHA78 highlighted that substantial progress has been made in Emergency, Critical, and Operative (ECO) care,^10^ which was proposed and implemented in accordance with the prior resolutions established by WHA77. The member countries are actively implementing and incorporating the ECO services into their national strategies, with a focus on sustainable financing and innovative healthcare infrastructures. It is also reported that the WHO’s new Acute Care Action Network (ACAN) is providing support to the ECO in low- and middle-income countries (LMICs). The continued focus on ECO care as a global priority would save millions of lives.^11^ This strategy is expected to guide countries in ensuring universal access to integrated emergency, critical, and surgical services. These are the vital components of health system resilience and emergency preparedness.

*Global Health Security:* It is anticipated that the WHA’s resolution will improve global health security through the Pandemic Agreement, along with effective functioning and stronger funding mechanisms. It is anticipated that the outcome of the WHA78 will be to enhance the WHO’s progress toward UHC, the creation of more climate-resilient health systems, and the empowerment of vulnerable communities through participatory health governance. The WHA78 decisions may be a significant step toward a more equitable and better-prepared global health landscape. This article aims to 1) examine the WHA78’s contributions to health promotion, equity, and reducing disparities, and 2) propose actionable strategies to navigate implementation challenges and emphasize the need for global solidarity to achieve health for all.

## Pandemic Agreement: Equitable Access to Health Resources

 The adoption of the Pandemic Agreement on May 20, 2025, is a historical step in pandemic history.^12^ It represented a landmark measure toward the promotion of health equity. Under this agreement, the WHA is mandated to allocate the WHO 20% of pandemic-related products, such as diagnostics, therapeutics, and vaccines, to address the needs of low-income countries. It is argued that this agreement can address the economic disparities, which are a key social determinant of health. This also aligns with the Ottawa Charter’s principle,^13^ which aims to create supportive environments by ensuring marginalized populations remain part of the inclusive healthcare system to address the global health crisis. It can encounter the inequities, for instance, considerable vaccine inequities during the COVID-19 pandemic, where wealthier nations hoarded resources. In this context, Usher noted that high-income countries used their substantial economic and political influence to secure a disproportionately large share of vaccine doses for their populations.^14^ Another study by Pushkaran et al. highlighted that the vaccine disparity experienced by the African continent was substantial, with merely 2.4% of its population vaccinated, in contrast to 41% of the North American population and 38% of the European population.^15^ Therefore, this Pandemic Agreement would be a significant step to foster health equity by providing accessibility to the vulnerable communities.

 On the contrary, the implementation of this agreement faces significant challenges due to the US withdrawal from the WHO (effective from mid-2025), limiting global cooperation, and the lack of enforceable mechanisms in the Agreement undermines its potential. This will also impact national health systems, which benefit from the WHO through guidelines, technical support, and data sharing on critical health issues such as vaccine development, antimicrobial resistance, and disease control programs (e.g., HIV/AIDS, polio, malaria). To operationalize ratifications of the Pathogen Access and Benefit Sharing (PABS) negotiations, at least 60 members must be ratified by July 2025. It is believed that it would be critical to effectively operationalize equity. Community-driven advocacy is another important dimension that can amplify marginalized voices, ensuring that global policies are translated into equitable outcomes at the grassroots level.

## Health Financing and UHC: Strengthening Equitable Systems

 The health financing resolution adopted by the WHA on May 24, 2025, was intended to address a projected 40% reduction in external health aid and rising out-of-pocket expenses (OOP). It is reported that OOP disproportionately burdens the low-income communities/countries. Globally, people in developing countries spend an estimated half a trillion dollars out of pocket each year on health care, according to the World Bank. In Sub-Saharan Africa, millions of people spend over 10% of their household consumption on health.^16^ By prioritizing the public health budgets along with the people-centered primary health care, the resolution of health financing advances the UHC, which is the cornerstone for health promotion and equity.^17^ The WHO’s approved US$4.2 billion budget for the period of 2026–2027, further supported by a 20% increase in assessed contributions, reflects the commitment to resilient, equitable, and affordable healthcare systems. It is aligning with the Ottawa Charter’s call for healthy public policy. The Assembly expressed the need to accelerate the process for equitable progress towards the UHC. Further, the WHA 78 approved resolutions to enhance national capacities, address rare illnesses as a global health priority for equity and inclusion, improve global health financing, and bolster medical imaging capabilities.^6^

 Conversely, notwithstanding these healthcare commitments, the WHO’s $600 million shortfall for 2024–2025, given by the several dynamics, is particularly worsened by the US withdrawal. It threatened the UHC’s progress. The WHA advocated for innovative financing, such as health taxes on alcohol, sugary beverages, and tobacco, etc. These taxes address NCD burdens while generating revenue for equitable health systems. Community-based campaigns can ensure that increased budgets prioritize underserved populations, reducing disparities in access to health care and equity. Empowering communities to demand accountable health financing aligns with health promotion’s participatory ethos and fosters equity at the systemic level. As Auerbach highlights, to protect the health of the population, the public health sector must establish robust partnerships with individuals and community-based organizations at high risk to promote equity.^18^ Numerous studies have highlighted the critical role of global health diplomacy in prioritizing equity in vaccine distribution,^19^ promoting digital health,^20^ advancing multilateralism,^21^ and improving access to medicines.^22^

## Protecting Vulnerable Populations: Nutrition and Equity

 WHA78’s resolution on regulating the digital marketing of breastmilk substitutes would strengthen protection against exploitative practices. It would empower the caregivers and infants with informed health choices.^23^ This resolution strengthens the community actions with the protection of the vulnerable populations. A recent multi-country study by Save the Children found 5183 instances of digital marketing that breached the International Code of Marketing for Breastmilk Substitutes (the Code). Furthermore, the Breast Milk Substitute industry employs social media marketing methods forbidden by the Code, including beginning direct contact with mothers, offering instructional materials through sponsored healthcare professionals, and making unproven health and nutrition claims.^24^

 Nutrition is significantly linked to noncommunicable diseases (NCDs) as dietary patterns influence NCD risk factors such as obesity, high blood pressure, high blood glucose, and abnormal blood lipids. Poor nutrition, particularly excessive intake of processed foods, sugar, and unhealthy fats, can contribute to chronic inflammation and insulin resistance, whereas poor nutrition, especially during childhood, is associated with a higher risk of developing NCDs later in life. The focus on NCDs and nutrition, linked to the Nutrition for Growth Summit, addresses the commercial determinants of health, such as the robust marketing of unhealthy products, and promotes equitable health outcomes.^25^ Advocacy for stricter marketing regulations ensures equitable access to health information and empowers vulnerable groups to make informed decisions. It is an important step toward health promotion equity. For example, Mexico, Barbados, and France all enforce national nutritional regulations and guidelines to address NCDs, with specific policies on food labeling, public health campaigns, and educational settings.^26^

 The equity-focused agenda and resolutions were passed by the WHA78’s which are likely to have significant impacts on health equity promotion, along with significant financial challenges. The withdrawal of the US would pose a challenge to the WHA resolutions, particularly regarding funding and global cooperation, as its budget has already been falling short. This financial shortfall limits the implementation of equity-focused resolutions. The geopolitical constraints further complicate its progress. The WHA78 has effectively underlined the health promotion equity, aligning with the Ottawa Charter principles. The WHA has prioritized addressing the social determinants, community empowerment, and equitable health access. The WHA78’s outcomes included the Pandemic Agreement, health financing, and nutrition protections by demonstrating the potential to reduce health inequalities. The WHO is likely to face challenges due to a $600 million shortfall. However, there are alternate ways to overcome these challenges through specific, effective implementation of community-driven strategies.

## A Way Forward

 To overcome these financial challenges and weak enforcements, the global health governance mechanisms should prioritize health disparities,^19^ strengthen cooperation & solidarity, promote innovation through digital health interventions, and focus on community empowerment. Advancing healthcare equity through technology requires healthcare professionals and policymakers to devise and execute intelligent healthcare systems that prioritize equity and accessibility.^20^ The equity can be further complemented by the enforceable compliance mechanisms of the Pandemic Treaty. The regional pharmaceutical manufacturing hubs can be stimulated by pooling local resources and by international cooperation with the WHO and the WHA. The ratification of the PABS system should be expedited. Secondly, in terms of financing, middle-income and emerging economies must take on a greater responsibility and leadership role in identifying and mobilizing resources to bridge the gap in leadership and funding. Further, funding shortfalls can be bridged through innovative financing tools such as health taxes, global health bonds, and domestic resource mobilization. These strategies can complement the WHO Investment Round for predictable funding. On the other hand, South–South cooperation (SSC) must be strengthened by sharing health technologies and extending financial support not only during emergencies (such as the recent COVID-19 pandemic), but also during normal times.

 The United Nations Office of SSC characterizes SSTC (South-South and Triangular Cooperation) as a comprehensive framework of collaboration involving two or more developing countries that share knowledge, skills, expertise, and resources to achieve their development objectives through coordinated efforts. Triangular cooperation involves collaboration in which conventional donor countries and multilateral organizations support South-South initiatives by providing financing, training, management, technological systems, and other forms of assistance.^27^ For example, the training of Nepali clinical trial personnel in India, along with the Indonesian, Nepali, and Filipino investigators conducting the clinical trials of a single vaccine in collaboration with the International Vaccine Institute, exemplifies a prominent instance of SSTC advancing vaccine development.^28^ Thirdly, the grassroots level of advocacy, investment in expansion and training of community health workers, and health literacy campaigns can create health equity for the marginalized sections of society. Addressing the commercial determinants of health through the stricter regulation of exploitative marketing, health equity policies, nutrition-sensitive policies, and climate-resilient health systems must be prioritized. Finally, digital health infrastructures and global data-sharing platforms must be shared and expanded to strengthen pandemic preparedness and health equity. By effectively governing and implementing these tools and strategies, we can advance health equity and realize the WHA78 vision of ‘One World for Health’.

## Conclusion

 WHA78 has advanced health equity by addressing the Pandemic Agreement, health financing reforms, and protections of vulnerable groups. Countries and States should practice health diplomacy and prioritize health disparities in their health agendas, strengthen cooperation between countries, promote innovation, and focus on community empowerment. The strategies to bridge inequities, strengthen the financing landscape, expand UHC, support south-south cooperation, empower communities, and promote innovation and digital health are very promising for shaping WHA 79 and future global health governance. It is imperative to implement strict enforcement mechanisms, mobilize and secure sustainable financing, empower communities, and maintain a consistent advocacy for equity to achieve the WHO’s vision of “One World for Health.”

## Competing Interests

 Vijay Kumar Chattu is an editorial board member of Health Promotion Perspectives, and the first author has no conflict of interest.

## Ethical approval

 Not Applicable
